# Driving guidelines and abdominal aortic aneurysms: a comparative analysis of regulations in Ireland and the United Kingdom, and other countries

**DOI:** 10.1007/s11845-026-04402-2

**Published:** 2026-04-24

**Authors:** Morgan Thomas McLoughlin, Nawar Masarani, Mohammed Elkassaby, Gergely Gosi

**Affiliations:** Department of Vascular Surgery, University Hospital Waterford, Dunmore Road, Waterford, Ireland

**Keywords:** Vascular surgery, AAA, Abdominal aortic aneurysm, Driving guidelines, Policy

## Abstract

**Introduction:**

Abdominal aortic aneurysms (AAAs) pose a significant health risk, particularly due to the potential for sudden rupture, which may lead to catastrophic consequences. It is hypothesised that rupture of an AAA may cause loss of consciousness whilst driving. Many countries have implemented regulations to govern driving eligibility for individuals diagnosed with AAAs, with significant international variation in criteria and enforcement.

**Aim:**

The aim of this paper is to examine the discrepancy in medical fitness-to-drive regulations in Ireland and the United Kingdom (UK) and compare to other international standards. An additional aim is to explore vascular guidelines and the scientific evidence for the aneurysm size threshold at which driving cessation is advised.

**Methods:**

Driving guidelines relating to AAA from selected international jurisdictions were reviewed and compared with Irish and UK standards. Guidelines were assessed for thresholds for notification, driving restriction, repair, differences between Group 1 and 2 drivers, relicensing criteria, and other relevant regulatory features.

**Results:**

The findings reveal significant international heterogeneity and a lack of standardisation in guidelines for Group1 drivers. Homogeneity is strong internationally for Group 2 drivers, and regulations enforce a 5.5 cm size threshold for driving disqualification, in keeping with current guidelines for surgical repair. The scientific level of evidence for these guidelines are limited, and weak where present.

**Conclusion:**

This current international guidance is disparate on Group 1 drivers and uniform for Group 2 drivers. This paper highlights the need for greater international consensus and justified driving policies to ensure both patient and public road safety.

## Introduction

Abdominal aortic aneurysms (AAAs) are defined as a focal dilation of the abdominal aorta to greater than 50% their normal diameter, or at least 3.0 cm in size [1, 2]. The primary risk associated with AAAs is rupture and subsequent death [3]. It is well recognised that rupture rates are significantly low below 5.5 cm, with modern consensus and analysis quoting less than a 0.4% annual risk [4]. It is widely accepted that the risk of rupture increases with aneurysm size. Previous randomised control trials (RCTs) have demonstrated no benefit to patient for a repair below a 5.5 cm threshold placing this size as the benchmark for elective intervention [5, 6]. General consensus exists that risk of rupture for AAA ≥ 5.5 cm outweighs the risks associated with peri-operative mortality, and great homogeneity is seen in this size recommendation for repair across all international vascular surgery guidelines [7, 8].

The concern over AAAs and driving chiefly pertains to the risk of sudden rupture and ensuing cardiovascular collapse, which could lead to a vehicle accident with fatal consequences for driver and other road users [9]. In relation to this concern, many government bodies and driving organisations have established regulations prohibiting driving for individuals with AAAs at certain thresholds. However, international consensus on these regulations vary significantly between countries for private drivers (Group 1), with some implementing stringent size-based restrictions and others relying on case-by-case physician assessments.

The evidence for AAA repair at 5.5 cm remains an area of ongoing debate. Data supporting the risk of rupture at this size are largely historic in nature and modern analysis from large population screening reveals that risk of rupture for small aneurysms is much smaller than previously speculated [10]. Questions, therefore, remain over the accuracy of historic data relating to larger aneurysms which currently influence present practice; Equally, similar questions relating to the overestimation of risk of rupture for large aneurysm (> 5.5 cm) are posited [11]. The decline in smoking rates and an uptake in prophylactic atherosclerotic best medical therapy are an additional compounding factor for the inapplicability of historic data to today’s modern population [12].

### Aims

This paper aims to: Compare and contrast current international driving guidelines for patients with AAAs; Examine the scientific basis supporting these regulations; Explore underlying reasons for international disparity in guidelines; And to present a need for international consensus and underlying reasoning for size-based thresholds.

## Methods

This study was designed as a narrative comparative review of publicly available driver-licensing regulations and guidance documents relating to AAA. Regulatory documents were identified through targeted web-based searches of national licensing/regulatory authority websites and publicly accessible professional guidance documents. Countries were included where English-language or readily translatable guidance was publicly available and contained explicit reference to AAA and driving.

In addition, major vascular surgery guidelines were reviewed to assess whether driving-specific recommendations were provided. This was not a formal systematic review of the medical literature or of all international licensing regulations, and jurisdictions without readily accessible guidance may, therefore, be underrepresented.

Driving regulations were analysed for data pertaining to: Driving regulations on abdominal aortic aneurysms; Requirements to notify a regulatory body; The corresponding size for notification; The size for prohibition of driving; Discrepancies for Group 1 and Group 2 drivers; Intervals for physician assessment; Policies in relation to relicensing; A rationale for driving prohibition; and any additional information.

For the purpose of this paper the evidence and analysis will be restricted to infrarenal AAAs of atherosclerotic origin and those which are fusiform in nature, where appropriate, and specific data is available.

## Results

**Table 1:** Comparative Analysis of International Driving Regulations

Country	AAA Size Restriction (Group 1)	AAA Size Restriction (Group 2)	Required Medical Notification	Notes
Ireland	≥ 6.5 cm	≥ 5.5 cm	Yes, ≥ 6.0 cm	License reinstated after successful treatment.
UK	≥ 6.5 cm	≥ 5.5 cm	Yes, ≥ 6.0 cm	License reinstated after successful treatment.
US	≥ 5.5 cm Men; ≥5.0 cm Women	≥ 5.5 cm Men; ≥5.0 cm Women	Yes, ≥ 4.0 cm	FMCSA Recommendations; Interstate variability
Canada	≥ 7.0 cm	≥ 5.5 cm	Yes	Size may be overruled if annual rupture risk < 20% and < 1%, respectively
European Union	Physician discretion	≥ 5.5 cm	Yes	Group 1 prohibited if size predisposes to a significant risk of sudden rupture
Australia	≥ 5.5 cm	≥ 5.5 cm	Yes; Requires Vascular Surgeon	Conditional licences; ≥5.0 cm prohibited for genetic aortopathy
Sweden	No specific size threshold	No specific size threshold	Yes	Driving advice is case-by-case.

*AAA* Abdominal aortic aneurysm, *FMCSA* Federal Motor Carrier Safety Administration

### Ireland

Ireland’s National Driver Licence Service (NDLS) has clear size-based restrictions informed by the Road Safety authority (RSA) [13]:

- **Group 1 (Car and Motorcycle) Drivers**: At a size of >6 cm diameter for AAAs, patients are permitted to drive subject to annual review, despite treatment. Driving is prohibited if the aneurysm reaches 6.5 cm.
- **Group 2 (Bus and Lorry) Drivers**: Driving is restricted if the aneurysm reaches 5.5 cm. Patients are permitted to drive following satisfactory medical or surgical treatment.

### United Kingdom (UK)

The UK’s Driver and Vehicle Licensing Agency (DVLA) has clear size-based restrictions [14]:

- **Group 1**: Patients must inform the DVLA if aneurysm is 6 cm or more in diameter despite treatment. Patients must not drive at a size of 6.5 cm or more.
- **Group 2**: Patients must notify the DVLA at any aneurysm size. Driving is prohibited if the aneurysm reaches 5.5 cm. Re-licensing may be considered post successful treatment.

### United States (US)

No unifying government body exists for recommendations or enforcement of guidelines on a national scale in the US for non-CMVs (Commercial Motor Vehicle) (Group 1). The Federal Motor Carrier Safety Administration (FMCSA) provides national US regulations for CMVs (Group 2). Interstate heterogeneity exists in the US with regards the governance of road users and, therefore, the provision of local guidelines and their enforcement.

- Group 1: Considerable heterogeneity exists between states with varying recommendations. There is a large theme of physician review of cardiovascular diseases and a threshold of 5.5 cm for driving disqualification.
- Group 2: Current FMCSA guidelines recommend that for CMV drivers that driving be prohibited at a size of greater to or equal than 5.5 cm for men and 5.0 cm for women [15]. AAAs < 5.5 cm, however, ≥ 4.0 cm, require annual physician review and clearance. The 2002 guidelines previously restricted driving of both sexes at a size of ≥5 cm, providing they were asymptomatic and were deemed fit by a physician [16].

## Canada

The Canadian Council of Motor Transport Administrators (CCMTA) is the regulatory body for driving in Canada. The CCMTA published regulations entitled ‘National Safety Code Standard 6: Determining Driver Fitness in Canada’ in 2021, which provides national guidance on AAAs and driving, as below [17].

- **Group 1**: Driving is prohibited at an AAA size of >7 cm diameter. Exceptions can be made if, in the opinion of the vascular surgeon, the annual risk of rupture is acceptable for non-commercial driving (generally less than 20%).
- **Group 2**: Driving is prohibited at a size of > 5.5 cm diameter. Exceptions can be made if, in the opinion of a vascular surgeon, the annual risk of rupture is acceptable for commercial drivers (generally less than 1%).

All individuals may be considered for relicensing once repair is performed and the treating physician (i.e. vascular surgeon) supports a patient return to driving.

Of note, the Canadian Medical Association (CMA) states in the 10th edition of ‘*Determining medical fitness to operate motor vehicles*’ that any aortic “aneurysm that warrants surgical intervention is inconsistent with operating a road vehicle” [9]. The recommendations state that the decision to license an individual with an AAA greater than size of threshold of repair should take into consideration the size and patient comorbidities.

### European Union (EU)

The European Working Group on Driving and Cardiovascular Disease acts as an advisory board to the Driving Licence Committee of the European Union. This working group published a report in 2013 entitled ‘*New Standards for Driving and Cardiovascular Diseases*’ [18]. These recommendations are intended to support uniformity across EU member states whilst allowing for clinical judgment in assessing individual risk and driving fitness. However, member states retain the authority to enforce stricter national standards, if desired.

- **Group 1 Drivers**: Driving licences will not be issued to, or renewed for, applicants or drivers if the maximum aortic diameter is such that it predisposes to a significant risk of sudden rupture and hence a sudden disabling event.

No specific size is provided and thus driving prohibition is left to physician discretion.

- **Group 2 Drivers**: Driving licences will not be issued to, or renewed for, applicants or drivers if the maximum aortic diameter exceeds 5.5 cm.

### Australia

Austroads is the collective transport regulatory body for both Australia and New Zealand. They provides clear size based regulations for driving in their ‘Assessing fitness to drive’ publication [19].

- **Group 1 Drivers**: Driving is prohibited at 5.5 cm for atherosclerotic AAAs. Driving is prohibited at 5.0 cm for patients with a genetic aortopathy.
- **Group 2 Drivers**: Driving is prohibited at 5.5 cm for atherosclerotic AAAs. Driving is prohibited at 5.0 cm for patients with a genetic aortopathy.

Individuals with AAAs are not permitted to hold unconditional licenses, and conditional licenses may be provided following periodic and annual review for non-CMV and CMV drivers, respectively. The ‘treating specialist’ (i.e. vascular surgeon) are required to provide information on size and aneurysm aetiology (atherosclerotic versus genetic aortopathy) for the fitness to drive process. Group 1 drivers may resume driving 4 weeks post repair. Group 2 drivers must wait 3 months.

### Sweden

No formal regulation exist with regards size based legislative criteria for Sweden. A medical certificate may be required to obtain a driving license or maintain an existing one if patients have a cardiovascular-related diagnosis. In these cases, a specialist doctor with relevant experience is required to issue the certificate.

### Additional countries

Information were not readily available for additionally searched countries, such as: China; Japan; South Korea; South Africa; Brazil; and Switzerland.

## Discussion

### Scientific basis for driving restrictions in AAA patients

Driving restrictions for patients with AAAs are largely precautionary in nature. There is no availability of data directly comparing AAA size to driving-related adverse events. The 5.5 cm threshold commonly used in operative decision-making originates from several large studies, which have greatly influenced current practice on AAA repair [5, 6]. It is implied that rupture whilst driving could result in devastating consequences for driver and other road users, and that the likelihood of this event increases with AAA size [9]. However, no prospective studies have addressed the incidence of AAA rupture whilst driving and, equally, no quantifiable risk of driving impairment due to AAA-related complications have been established.

Heterogeneity is certainly evident in inter-country regulations on AAAs and driving. In the absence of robust empirical data, the driving guidelines across the globe remain largely speculative rather than evidence-informed. Observational and epidemiological studies on AAA size and driving safety are lacking and further research is required to identify an appropriate AAA size for driving prohibition, with international consensus.

### Rationale for precautionary driving bans

In the absence of compelling causative data, it is implied (where absent) that international regulatory bodies on driving justify licensing restrictions for AAAs based on the intention of minimising risk to road users. Stringent regulations are clearly evident for Group 2 road drivers, strengthening this association with attempted risk minimisation, as a public health strategy. The primary concern of increased AAA size is rupture, and an ensuing cascade of acute hypotension, loss of consciousness, or even sudden death, which could pose a catastrophic danger to driver and other road users. Furthermore, symptomatic AAAs without rupture, may cause symptoms of acute abdominal pain, dizziness, or pre-syncopal events which could impair driver reaction time and decision-making while driving.

International uniformity is observed for CMVs in Table 1, and this directly correlates with current international guidelines on a 5.5 cm size for repair (and 5.0 cm size of repair for women, where considered). It is therefore assumed, although not always stated in driving regulations, that similar to guidelines for repair, the annual risk of rupture at a size of 5.5 cm is of a significant threat to life to warrant public health regulatory measures.

There is a clear absence of underlying rationale accounting for each regulatory body’s decisions for both Group 1 and Group 2 driver prohibition. The risk of AAA adverse events whilst driving is assumed to be larger for Group 2 drivers, who may be carrying passengers or heavy loads. It is hypothesised that this concern influences the homogeneity in driving regulations from all guidelines in this review. Despite this inferred rationale accounting for more stringent regulations for CMVs, it is not clearly stated in any of the above regulatory guidelines.

Current international regulatory bodies providing threshold sizes for repair greater than 5.5 cm are operating against current standards of indications for repair and, largely, without justification. Greater explanation is required from individual regulatory bodies with regards their tailored size threshold strategies to provide insights into regulation, and to influence patient safety. An examination and collaboration of international legislation may allow for consensus. Notably, our review of major contemporary aortic guidelines identified very limited guidance on AAA and driving regulation. The European Society for Vascular Surgery (ESVS) 2024 guidelines on the Management of Abdominal Aorto-Iliac Artery Aneurysms does not currently provide any recommendations on driving and AAA size regulations [7]. Given the policy variability identified in this review, major vascular guideline organisations, such as the ESVS, could usefully provide overarching recommendations on fitness to drive in patients with AAA. Such recommendations would not be intended to replace national licensing frameworks, but rather to provide a common evidence-informed reference point from which individual countries could adapt guidance within their own legal and regulatory systems. A central recommendation of this kind could help facilitate greater standardisation, improve transparency, and clarify the rationale underpinning national policies. However, any such effort must also acknowledge the important limitations of the current evidence base: contemporary data on AAA rupture risk remain imperfect, and further to this review, we identified no studies or case reports directly examining AAA rupture while driving, AAA-related driving death, or loss of vehicle control attributable to an AAA event.

It is also important to recognise that the issues highlighted here are not unique to AAA or to cardiovascular disease. More broadly, the assessment of medical fitness to drive is inherently challenging across multiple bodily systems, particularly where regulators and clinicians must distinguish between Group 1 and Group 2 drivers, balance individual mobility and livelihood against public safety, and translate limited or imperfect condition-specific evidence into practical licensing rules. Variability in thresholds, reliance on clinician judgement, and differences in the regulation of private and commercial drivers are, therefore, not exclusive to AAA, but reflect wider difficulties within medical driver licensing, of which AAA is one illustrative example.

### Variability in international regulations

The countries which yielded results for this review were largely western nations with a strong demographic of Caucasian populations. Aortic aneurysm prevalence varies significantly throughout the globe and is influenced by race, genetic factors, and lifestyles. Western populations, particularly those in Europe, North America, and Australia, report higher AAA prevalence due to increased risk factor association. In contrast, studies suggest that AAA prevalence is substantially lower in Asian, African, and South American populations. These geographic and demographic discrepancies may have influenced the over representation of AAA specific guidelines in western countries and explain why data were not available for Asian, African, and South American nations. It is proposed that amongst these nations public health measures have been prioritised on other disease burdens.

The emphasis on AAA pathology in western nations is underscored by the presence of structured AAA screening programmes. These formal, national programmes exist for the US, the UK, Italy and Sweden, and are not present for other nations. Despite a clear emphasis on AAAs as a pathology of public health concern, no size-based criteria legislation has been introduced for Sweden and cases are reviewed by physicians on an individual basis. It is, however, likely that Swedish based decisions are influenced by the EU working group recommendations in the absence of national legislation.

The extent of discrepancy within Europe may also be less pronounced than the broader international variation identified in this review. While national differences remain, the European position is partly shaped by an existing supranational framework, whereby EU guidance provides general principles and allows member states to exercise discretion or adopt stricter national rules. As such, the greatest heterogeneity identified in this review appears to relate more clearly to the international comparison overall, and particularly to Group 1 drivers, than to variation within Europe alone.

### A need for consensus

An interesting result from the review of the driving guidelines is the permission of driving at sizes above the threshold for repair. Current legislation for Group 1 drivers permits driving in some nations up to 6.5 and 7.0 cm, which appear to lack collaboration with current international practices of a 5.5 cm size for elective repair for AAAs. Taking current guidelines into consideration one would expect uniformity with current surgical intervention recommendations. In the absence of regulatory bodies proving underlying rationales for driving at size thresholds above repair, the current regulations appear unjustified.

This is echoed by the Canadian CMA advice that states that an “aneurysm that warrants surgical intervention is inconsistent with operating a road vehicle” [9]. This statement presents irony as Canadian regulatory guidelines for Group 1 drivers are amongst the most lenient guidelines across the globe (> 7.0 cm). The Canadian regulatory body, however, is amongst one of few government organisations who have provided a rationale for the size threshold of driving restriction. A 7 cm size for Group 1 drivers is provided, quoting an annual 20% risk of rupture as the upper limit of acceptability for private drivers. This provides insights into the current thought processes influencing AAA size thresholds and driving in Canada. Despite providing an explanation, the underlying rationale, however, for this size threshold must be scrutinised. There is no clear scientific evidence provided which justifies the appropriateness of this level of risk, and one has to question the acceptability of 20% as a value for risk of rupture and driving. A rationale of a risk of rupture of less than 1% is provided for Group 2 drivers, and again, justification for this level of risk is unexplained.

Further justification is clearly required to validate these size thresholds.It is unlikely that formal medical fitness-to-drive regulations can directly incorporate all of the personal, occupational, and economic consequences of driving disqualification for individual patients, as such frameworks are primarily intended to provide practical and safety-focused licensing thresholds. Nevertheless, these considerations may still be relevant at the level of broader policy development, particularly when regulators seek to ensure that restrictions are proportionate to the risk posed. In this context, greater transparency regarding the rationale underpinning threshold selection would be valuable, even where wider social and economic factors cannot be explicitly integrated into the regulatory criteria themselves.

Interestingly, for Group 1 drivers, the EU working group recommends prohibition of driving at a size which predisposes a driver to a significant risk of rupture and consequent disabling event. The UK guidelines characterise the risk of a sudden disabling event as: 20% likelihood of an event in 1 year for Group 1 licensing; and 2% likelihood of an event in 1 year for Group 2 licensing, however, this a general definition, and not AAA-specific. The Irish guidelines are more tenuous and do not quote an acceptable level of annual risk. However, inferring data from driving restrictions on seizures in this Irish guideline, the NDLS similarly quote an acceptable annual risk as 20% for Group 1 and 2% for Group 2 drivers, respectively. Neither guideline specifically justifies this statement for AAA driving restrictions. Despite the lack of rationale, upper limits for AAAs and driving of 6.5 cm are provided.

### Are current driving guidelines progressive?

An additional factor to consider is a current emphasis in the literature towards extending the size threshold of AAA repair above the current 5.5 cm guidelines. Observational studies in the last decade on large AAAs unsuitable for elective repair have shown rates of rupture lower than previously anticipated, despite an overall high mortality in this cohort [20]. There is a dearth of well powered trials investigating the conservative management of aneurysms above a 5.5 cm threshold. The only RCT in publication to address this clinical question is the EVAR 2 trial, which notably shows no significant difference in overall survival between groups [21]. The study’s cohort (1999–2003) is archaic with regards to today’s risk modified population, lending further support to the hypothesis of continued patient safety with an increased AAA threshold size for repair. Furthermore, contemporaneous publications using sensitivity analyses on maximum AAA rupture risk have provided data to suggest the safety of extending the safe threshold size of repair to 7 cm in some populations [22].

Modern perspectives on AAA size threshold for intervention are shifting towards increased diameters, and they may provide an evidence basis and the consequent rationale of permissible safe driving at a larger aneurysm size (up to 7.0 cm). However, it is worth noting, that whilst revised, most regulatory guidelines have remained unchanged over the decades, and their recommendations predate the modern shift towards an increased AAA size repair threshold.

Clearer guidance is also required for justifying physician discretion for driving permission, if applied, and may require more factors than calculating a patient specific risk of rupture, and include holistic factors such as detriment to patients’ quality of life. International consensus is clearly missing, and further data is required and international collaboration to provide a clear rationale and justification for AAA size disqualification.

## Conclusion

Guidelines for AAAs and driving for Group 1 drivers are disparate, however, currently align with modern perspectives on AAA size repair thresholds, despite largely predating them. International unity for a 5.5 cm size threshold for Group 2 drivers exists and this consensus correlates with an implied greater risk of catastrophic events for CMVs. Group 2 driving regulations align with current global guidelines on a 5.5 cm size threshold for AAA repair.

Prospective data on AAAs and driving is required to assess the clinical safety of driving at different size thresholds. Ultimately, data is required on the prevalence of AAA adverse events and driving to draw meaningful conclusions on recommendations for driver disqualification. It is important for international driving regulatory bodies to provide a rationale for AAA size of disqualification which may explain current regulatory discrepancies.
